# The Landscape of Randomised Controlled Trials of Therapies in Breast Cancer in Low and Middle Income Countries

**DOI:** 10.1155/2017/4259704

**Published:** 2017-04-09

**Authors:** Katharine Lynch-Kelly, Matthew Singer, Norman R. Williams

**Affiliations:** Surgical & Interventional Trials Unit, Division of Surgery and Interventional Science, Faculty of Medical Sciences, University College London, London, UK

## Abstract

*Objectives*. The objectives of this study were to identify the randomised controlled trials in breast cancer occurring in low and middle income countries (LMICs) generally and within Sub-Saharan Africa specifically, to describe the current status and identify opportunities for further research in these areas.* Materials and Methods*. Data for this study were obtained from ClinicalTrials.gov. The search term “Breast Cancer Research” was used, and relevant information extracted and analysed.* Results*. 2414 trials were identified, of which 1099 were eligible for inclusion. 69 of these trials occurred in LMICs. Of the 52 LMICs globally, 30% were participating in breast cancer research. Of the 17 LMICs in Africa, 77% are situated in Sub-Saharan Africa; 23% were participating in breast cancer research, which accounted for 9% of total Sub-Saharan African studies.* Conclusion*. This study provides current evidence for the need for breast cancer research in LMICs globally and within Sub-Saharan Africa. Within LMIC regions where research is active, the type and numbers of studies are unevenly distributed. High quality research within such areas should be encouraged as the results may have both local and global applications, particularly in the provision of affordable health care.

## 1. Introduction

**Figure 1:**
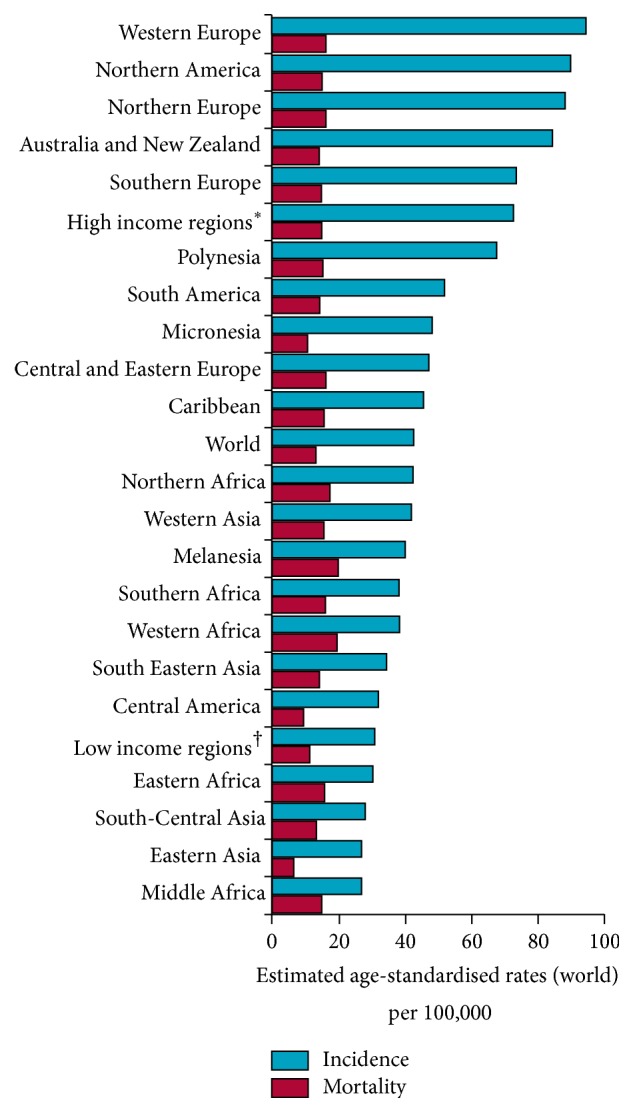
Global breast cancer incidence and mortality in women in 2012. Estimated age-standardised rates for breast cancer incidence (blue bar) and mortality (red bar) in females are shown for global regions, high income regions, low income regions, and the world. Reproduced* with permission* from Abenaa M Brewster, Mariana Chavez-MacGregor, and Powel Brown. *∗* includes all regions of Europe, Northern America, Australia, New Zealand, and Japan. † includes all regions of Africa, Asia (excluding Japan), Latin America, the Caribbean, and Federated States of Micronesia.

**Figure 2:**
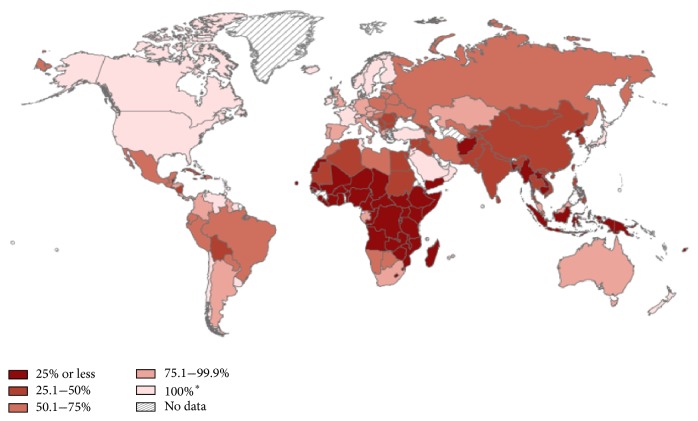
Global cancer statistics regarding the estimated percentage of patients with access to radiotherapy, 2012. Reproduced with permission from Volume 65, Issue 2, pages 87–108, 4 FEB 2015. *∗* refers to countries where 100% of the population can access radiotherapy or countries where radiotherapy is greater in supply than demand, despite possible regional differences in access to radiotherapy within the country. Source: The Cancer Atlas, second edition, as obtained from the International Atomic Energy Agency.

In 2012, women from less developed countries were less than half as likely to develop breast cancer by the age of 75 compared to those from more developed countries (8% v 3.3%), yet almost just as likely to die from breast cancer (1.6% v 1.2%) (see Figures 1 and 2) [1]. Safe and effective therapies continue to be developed; however their escalating cost means they are increasingly becoming out of reach of the patients who need them. Howard et al. [2] emphasised this trend by calculating the average launch price of anticancer drugs from 1995 to 2013, adjusted for inflation and health benefits. They found an increase of 10% annually—an average of $8,500 per year. Considering that it is estimated that by 2020 70% of the twenty million new cancer cases will occur in countries that collectively have only five percent of the global resources for cancer control [3], this trend will have global repercussions. Lower to middle income countries (LMICs) will be at the epicentre of the eruption that occurs when the high cost of treatment, coupled with increasing incidence rates, collides with a lack of access and ability to pay, unless suitable alternatives can be found. Such alternatives have to provide affordable, simple, and widely accessible solutions. Furthermore, in order to achieve global equality in long term breast cancer incidence and mortality, more evidence-based research addressing particular circumstances in LMICs themselves is required. This will generate the highest level of evidence in breast cancer research in LMICs to be produced. Factors influencing incidence, access to treatment, and mortality are to be identified and solutions formulated and scrutinised based upon these constraints, rather than continuing the expensive and evidence-poor culture of implementing effective treatment methods identified in high income countries (HICs) [4].

We have prepared an overview of randomised controlled trials currently being run in LMICs, in order to identify areas where further research is needed.

## 2. Materials and Methods

A descriptive analysis was performed of all of the trials with one or more locations in an LMIC. Data was extracted from ClinicalTrials.gov, a publicly available database of clinical trials [5]. On 29 August 2016 the search term “Breast Cancer Research” was applied to the 223,969 trials registered on ClinicalTrials.gov. The search results were downloaded onto a spreadsheet for tabulation and analysis. Trials with a withdrawn status and those that did not specify that they were randomised were excluded.

LMICs were grouped into the geographical regions South Asia, South East Asia, East Asia, Middle East, North Asia, Pacifica, Africa, South America, Central America, North America, and Europe as defined by clinicaltrials.gov, the US Congress definition of Sub-Saharan Africa was used (Table 1). South Sudan was not distinguished from Sudan in the clinicaltrials.gov database. For regional analysis, each region was selected on the website and the studies for each LMIC within a region were downloaded. Withdrawn trials and trials that did not specify a randomised status were excluded. Trials were crosschecked using NCT numbers to remove duplicates (trials listed twice or those which occurred in more than one LMIC).

Intraregional analysis, using the spreadsheets described above, was conducted on regions with a large number of trials occurring in that region, to identify intraregional distribution of trials within LMICs. Duplicate entries were removed from the number of trials tabulated within each LMIC in South Asia, South East Asia, North Asia, and Africa. For interventional analysis, the regional spreadsheets were used to determine the number of trials using each intervention. The data extracted for each trial included the recruitment status, study results, conditions, interventions, sponsor/collaborators, gender, age groups, phases, enrolment, source of funding, study type, first received, start date, completion date, results first received, and primary outcome.

## 3. Results

Of the initial 2414 trials, 1099 fit the criteria for inclusion in the analysis. Trials that did not specify that they were randomised or that had the recruitment status “withdrawn” were excluded. 1315 trials were excluded. Trial names and ClinicalTrials.gov identification numbers of all included trials are listed in the Appendix.

### 3.1. Global Results (Tables 2 and 3)

Of the 1099 trials included in the analysis, 69 (6.3%) occurred in one or more LMICs. Lower to middle income countries were identified 104 times for these 69 trials.

Of the 52 LMICs identified by the World Bank (Table 1), 30.8% (16) had breast cancer research located within them. 5 (9.6%) are located in South Asia, 7 (13.5%) are located in South East Asia, 1 (1.9%) is in East Asia, 3 (5.8%) are located in the Middle East, 6 (11.5%) are located in North Asia, 7 (13.5%) are in Pacifica, 17 (32.7%) are in Africa, 1 (1.9%) is in South America, 4 (7.7%) are in Central America, and 1 (1.9%) is in Europe (Table 2). No LMICs are located in North America.

The percentage of LMICs that contained active breast cancer research in each region was identified as 60% in South Asia (3), 42.9% (3) in South East Asia, 0% in East Asia, 0% in the Middle East, 16.7% (1) in North Asia, 0% in Pacifica, 35.3% (6) in Africa, 100% (1) in South America, 50% (2) in Central America, and 0% in Europe (Table 3).

### 3.2. Intraregional Analysis

35 breast cancer research trials were located at 37 sites amongst the 3 LMICs of South Asia (India, Pakistan, and Bangladesh). Of the 37 sites, 34 (91.9%) were located in India, 2 (5.4%) were located in Pakistan, and 1 (2.7%) was located in Bangladesh. 12 breast cancer research trials were located at 16 sites amongst the 3 active LMICs in South East Asia (Philippines, Vietnam, and Indonesia). Of the 16 sites, 12 (75%) were located in the Philippines, 2 (12.5%) in Vietnam, and 2 (12.5%) in Indonesia. 25 breast cancer research trials were located at 25 LMIC sites in North Asia; all 25 were located in Ukraine.

15 breast cancer research trials were located at 21 sites amongst the 6 active LMICs of Africa (Morocco, Tunisia, Egypt, Kenya, Ghana, and Nigeria). Of the 21 sites, 3 were located in Morocco (14.3%), 1 was in Tunisia (4.8%), 12 were in Egypt (57.1%), 1 was in Kenya (4.8%), 1 was in Ghana (4.8%), and 3 were in Nigeria (14.3%).

### 3.3. LMIC Breast Cancer Research Intervention (Table 4)

Intervention is categorised by clinicaltrials.gov as radiation, biological, behavioural, drug, other, or combinations of these options. For LMICs in each region, breast cancer research using drug intervention was the most common, representing at least 33.3% of the total breast cancer research in each region alone, and in combination with other categories; research containing a drug intervention represented 100% of the research in South East Asia and North Asia and 67% of the total breast cancer research in Central America, whilst only 20% of African breast cancer research on LMICs was not involving a drug intervention. Regions with the largest numbers of breast cancer research trials occurring in LMICs (Africa, South Asia, and North Asia) exhibited intervention with a large focus around the drug intervention type (60.0%, 68.6%, and 65.4%, resp.) with all other intervention types evenly distributed at a low frequency. Regions with fewer LMIC breast cancer research trials (Central America and East Asia) exhibited a more even distribution of intervention type; however this was still heavily focused around the drug intervention type. South America exhibited no drug or associated drug intervention, with 100% of its intervention focused on device and other intervention.

### 3.4. Sub-Saharan Africa

Of the 44 total countries that make up Sub-Saharan Africa, 4 (9%) have studies located within them (Nigeria, Kenya, Ghana, and South Africa).

Of the 17 LMICs in Africa (Table 1), 13 (76.5%) are located in Sub-Saharan Africa (Table 1); 3 of these countries (23.1%) contain breast cancer research.

Of the total 54 studies located at sites in Sub-Saharan Africa, 5 (9.3%) are located in LMICs, with the remaining 49 (90.7%) located in South Africa.

Of the 6 LMICs where research is undertaken in Africa, 60% (3) are located in Sub-Saharan Africa. They account for 23.8% (5) of the total LMIC sites in Africa, and 4 (26.6%) of the total LMIC studies in Africa. The remaining three African LMICs are located in North Africa (Egypt, Tunisia, and Morocco). These accounted for 16 (76.2%) of the total sites, and 11 (73.3%) of the total studies in LMICs in Africa.

## 4. Discussion

Our results show that, of the 1099 trials included in the analysis, 70 (6.4%) occurred in an LMIC, across 104 LMIC sites. Studies are unevenly distributed amongst regions: less than one-third of LMICs are undertaking breast cancer research globally, and in the Middle East, Pacifica, East Asia, and Europe there is no breast cancer research occurring in LMICs, despite Pacifica accounting for 13.5% of the world's LMICs.

Although some regions, such as South Asia, have a high percentage of breast cancer research in LMICs (60%), there is evidence that this is monopolised by one country. For example, 92% of the current breast cancer research studies located in LMICs in South Asia are undertaken in India where the healthcare expenditure per capita for 2012 is approximately double (1.7 and 2.2 times) that of Pakistan and Bangladesh, respectively [1]. A similar situation was found in Africa, where three North African LMICs (Egypt, Tunisia, and Morocco) are collectively responsible for 76% (16 of 21) of the total LMIC studies located in Africa.

Despite making up 44 of the 51 African countries, Sub-Saharan Africa is hugely lacking in breast cancer research. Sub-Saharan LMICs currently account for only 9.3% of the total studies located in Africa. Equally, only 4 (9%) of all Sub-Saharan African countries are undertaking breast cancer research trials. Furthermore, the distribution of total research within Sub-Saharan Africa is distorted; for example, South Africa undertakes 91% of the total 54 Sub-Saharan African studies, despite having a breast cancer mortality rate 2.1 times that of Kenya, 4 times that of Ghana, and less than one-third (29%) that of Nigeria, and a health care expenditure per capita for 2012 15.5 times that of Kenya, 7.6 times that of Ghana, and 7 times that of Nigeria.

As identified by Elzawawy [6], national cancer control programs may be present in LMICs; however they are global solutions implemented in countries with local challenges, causing limited accessibility and affordability. Chemotherapeutic-protocols from high income countries cannot be used in low income countries due to the lack of supportive care facilities and trained personnel. Less intensive, less toxic, and less expensive protocols are needed [7]. Equally, radiotherapy is highly inaccessible to most patients in Africa. Access averages 0.89 machines per million inhabitants compared to 8.9 per million in high income countries [8]; 29 out of 52 African countries have no facilities at all [9].

As highlighted by Elzawawy [6], clinical trials in LMICs take place within the economic, social, and logistical constraints to healthcare found in these regions, which means that research outcomes from trials here are applicable to populations facing similar challenges in high income countries. At a local level, research would enable local challenges to be identified and specific, realistic interventions to be designed. Research in LMICs could help to ensure global targets are met, such as the 80% availability of affordable basic technologies and essential medicines set by the WHO for 2020.

The repurposing of medications could also help to reach these targets, by providing a simple, cheap, effective alternative that is scientifically robust as well as realistic in the treatment of breast cancer. For example, the analgesic ketorolac, a relatively inexpensive nonsteroidal anti-inflammatory drug, given perioperatively is associated with a significantly superior disease-free survival in the first few years after surgery [10]. A simple treatment such as this would also be applicable to populations who, despite living in HICs, cannot afford therapies that are more expensive. Akinyemiju et al. [11] found that more than half of the African-American women diagnosed with breast cancer studied received no radiation or surgery as the first course of treatment despite 35% of cases being diagnosed at stages III or IV.

Furthermore, regions such as Africa can provide large groups of patients with a high prevalence of specific subtypes and stages of breast cancer for research and clinical trials. These populations are less prevalent in HICs, making studies in these populations very difficult and expensive. The research outcomes from such studies could be hugely beneficial to women worldwide. For example, African female populations present a higher proportion of triple-negative, hormone receptor negative tumours at a later stage. Research in these populations could provide a better understanding of type-specific cancers but also have applications in HICs with large populations of African ancestry. For example, in the USA, the lifetime risk of African-American women developing triple-negative breast cancer is almost double that of other races [11] and accounts for 20% of the breast cancer mortality rate in the USA despite only a 12% prevalence.

As discussed by Akinyemiju et al. [11], clinical trials conducted in LMICs need to have clear guidance regarding ethical conduct. Such trials must demonstrate an ethical control arm and maintain a local aim, with the potential of global extrapolation. With this in mind, it is ethical for the control arm to be placebo-based, so that potential value treatments are compared to no treatment rather than the ideal treatment, which by definition is too expensive and logistically convoluted to be accessed in the local community. It is crucial that this methodology for testing potential treatments must only be used if such treatments can realistically be implemented in the testing community. However, to maintain long term viability, trials in LMICs must be appropriately balanced between being ethical, beneficial to those undertaking them and the wider community and yet realistic in these aims, to maintain economic viability for the sponsors.

Greater research capacity in LMICs would also benefit from and enable the formation of long term research collaborations between institutions in HICs and LMICs. Such collaborations have been successfully established and maintained in other research areas such as the Harvard University and Universite' Cheikh Anta Diop of Dakar, Senegal collaboration regarding HIV/AIDS, and are beneficial to both HICs and LMICs, HICs providing greater access to grant funding, and models for research and infrastructure for LMICs, which in turn provide regional disease experience and knowledge, grant access, experienced public health officials, alternative demographics, and affordable treatments for HICs [12]. Greater breast cancer research in LMICs would provide the opportunity for such collaborations between LMICs and HICs to take place, stimulating both further research in LMICs, and the production of greater clinical and translational research, which has the potential to enhance global understanding of breast cancer and, crucially, provide affordable treatments.

## 5. Conclusions

Clearly there is insufficient research in LMICs globally. Less than one-third of LMICs participate in randomised controlled trials of breast cancer research; increasing the research undertaken in LMICs is fundamental if affordable, simple, and widely accessible healthcare treatments are to be discovered. Research in such regions will benefit the countries themselves by providing realistic treatment solutions that work around the constraints currently facing them. It can also provide affordable options to people globally who struggle to access treatments, which are rapidly increasing in price and complexity, making them an unrealistic and inaccessible option. In addition, LMICs enable research into specific subtypes of disease presenting at later stages. LMICs provide a research environment which, if harnessed, could produce solutions to not only the healthcare problems faced in these regions, but problems faced by people globally.

## Supplementary Material

List of trials from ClinicalTrials.gov used in this study.

## Figures and Tables

**Table 1:** *Lower-middle income economies as defined by the World Bank*. LMICs are categorized regionally based upon clinicaltrials.gov regional definitions. Countries written in italic font participate in RCTs in breast cancer research. Countries denoted with *∗∗* are members of Sub-Saharan Africa (US Congress definition).

Africa	South East Asia	East Asia
Cabo Verde^*∗∗*^	*Indonesia*	Mongolia
Cameroon^*∗∗*^	Lao PDR	
Congo, Rep.^*∗∗*^	Myanmar	Middle East
Cote d'Ivoire^*∗∗*^	*Philippines*	Syrian Arab Republic
Djibouti^*∗∗*^	* Vietnam*	West Bank and Gaza
*Egypt, Arab Rep.*	Timor-Leste	Yemen, Rep.
*Ghana*^**∗∗**^	Cambodia	
*Kenya*^**∗∗**^		Pacifica
Lesotho^*∗∗*^	South Asia	Kiribati
Mauritania^*∗∗*^	*Bangladesh*	Micronesia, Fed. Sts.
*Morocco*	Bhutan	Papua New Guinea
*Nigeria*^**∗∗**^	*India*	Samoa
Sao Tome and Principe^*∗∗*^	*Pakistan*	Solomon Islands
Sudan^*∗∗*^	Sri Lanka	Vanuatu
Swaziland^*∗∗*^		Tonga
Tunisia	South America	
Zambia^*∗∗*^	*Bolivia*	Europe
		Kosovo
North Asia	Central America	*Ukraine*
Armenia	*El Salvador*	
Kyrgyz Republic	*Guatemala*	
Tajikistan	Honduras	
Uzbekistan	Nicaragua	
Moldova		

**Table 2:** *The proportion of global LMICs per region*. The proportion of LMICs in each region out of the total number of LMICs globally (52) was calculated to determine the distribution of LMICs globally.

Regions	Total regional LMICs	Proportion of global LMICs per region (%)
South Asia	5	9.6
South East Asia	7	13.5
East Asia	1	1.9
North Asia	6	11.5
Africa	17	32.7
South America	1	1.9
Central America	4	7.7
Europe	1	1.9
Middle East	3	5.8
Pacifica	7	13.5

**Table 3:** *The proportion of breast cancer research active LMICs per region*. The proportion of LMICs in each region undertaking breast cancer research was calculated to determine the distribution of research within each region.

Regions	Active research LMICs	Active research LMICs within region (%)
South Asia	3	60.0
South East Asia	3	42.9
East Asia	0	0.0
North Asia	1	16.7
Africa	6	35.3
South America	1	100.0
Central America	2	50.0
Europe	0	0.0
Middle East	0	0.0
Pacifica	0	0.0

**Table 4:** *Types of intervention for RCTs in breast cancer research in LMICs in each region*. The percentage for each intervention was calculated. North America and East Asia are not included due to no LMICs being present in these regions.

Intervention	South America (%)	Central America (%)	North Asia (%)	South East Asia (%)	South Asia (%)	Africa (%)
Total number of studies	1	3	25	12	35	15
Procedure	0.0	0.0	0.0	0.0	0.0	6.7
Radiation	0.0	0.0	0.0	0.0	2.9	13.3
Other	0.0	0.0	0.0	0.0	2.9	0.0
Biological	0.0	0.0	3.8	0.0	2.9	0.0
Behavioural	0.0	0.0	0.0	0.0	2.9	0.0
Drug	0.0	33.3	65.4	58.3	68.6	60.0
Drug and radiation	0.0	0.0	0.0	0.0	2.9	0.0
Drug and procedure	0.0	0.0	11.5	33.3	2.9	13.3
Drug and procedure and radiation	0.0	0.0	0.0	0.0	2.9	0.0
Drug and procedure and other	0.0	0.0	0.0	0.0	2.9	0.0
Drug and Other	0.0	33.3	7.7	8.3	2.9	0.0
Drug and biological	0.0	0.0	11.5	0.0	5.7	6.7
Drug and behavioural	0.0	0.0	0.0	0.0	0.0	0.0
Procedure and biological	0.0	33.3	0.0	0.0	0.0	0.0
Device and other	100.0	0.0	0.0	0.0	0.0	0.0

## Appendix

List of trials from ClinicalTrials.gov used in this study (see Supplementary Material available online at https://doi.org/10.1155/2017/4259704).
