# Supplementary material for: The Landscape of Randomised Controlled Trials of Therapies in Breast Cancer in Low and Middle Income Countries
Source: Int J Breast Cancer. 2017 Apr 9;2017:4259704. doi: 10.1155/2017/4259704 (PMC5401736; doi:10.1155/2017/4259704)
Supplement: Supplementary file 1 — List of trials from ClinicalTrials.gov used in this study. [file 4259704.f1.docx]

# **Appendix**

ClinicalTrials.gov trials used in this study

| NCT02429427 |
| --- |
| NCT02393833 |
| NCT02352779 |
| NCT02338310 |
| NCT02297412 |
| NCT02278900 |
| NCT02224261 |
| NCT02170415 |
| NCT02154776 |
| NCT02152462 |
| NCT02150148 |
| NCT02137837 |
| NCT02116530 |
| NCT02104830 |
| NCT02058615 |
| NCT02042443 |
| NCT02015806 |
| NCT02004834 |
| NCT01942135 |
| NCT01940016 |
| NCT01913990 |
| NCT01910350 |
| NCT01907880 |
| NCT01900730 |
| NCT01881230 |
| NCT01876238 |
| NCT01874184 |
| NCT01867866 |
| NCT01835158 |
| NCT01822314 |
| NCT01800422 |
| NCT01783444 |
| NCT01764022 |
| NCT01741883 |
| NCT01740427 |
| NCT01712009 |
| NCT01705691 |
| NCT01688973 |
| NCT01658943 |
| NCT01647607 |
| NCT01642251 |
| NCT01637103 |
| NCT01627366 |
| NCT01613430 |
| NCT01610284 |
| NCT01609790 |
| NCT01602380 |
| NCT01594398 |
| NCT01593020 |
| NCT01579552 |
| NCT01572727 |
| NCT01557478 |
| NCT01547741 |
| NCT01530607 |
| NCT01527487 |
| NCT01515800 |
| NCT01515124 |
| NCT01508273 |
| NCT01504789 |
| NCT01498289 |
| NCT01492101 |
| NCT01485926 |
| NCT01479244 |
| NCT01478477 |
| NCT01452399 |
| NCT01446159 |
| NCT01434342 |
| NCT01421472 |
| NCT01411787 |
| NCT01381874 |
| NCT01376349 |
| NCT01355393 |
| NCT01349322 |
| NCT01346761 |
| NCT01306032 |
| NCT01299038 |
| NCT01296893 |
| NCT01293032 |
| NCT01275677 |
| NCT01266642 |
| NCT01237067 |
| NCT01231204 |
| NCT01224678 |
| NCT01219075 |
| NCT01204125 |
| NCT01198158 |
| NCT01191580 |
| NCT01163682 |
| NCT01160718 |
| NCT01151046 |
| NCT01149083 |
| NCT01131195 |
| NCT01104935 |
| NCT01101438 |
| NCT01098955 |
| NCT01097642 |
| NCT01097278 |
| NCT01091428 |
| NCT01086605 |
| NCT01084057 |
| NCT01081262 |
| NCT01077154 |
| NCT01074970 |
| NCT01073865 |
| NCT01068522 |
| NCT01064635 |
| NCT01049425 |
| NCT01017601 |
| NCT01015833 |
| NCT01009918 |
| NCT01008150 |
| NCT01007942 |
| NCT00999804 |
| NCT00981305 |
| NCT00976573 |
| NCT00976131 |
| NCT00971009 |
| NCT00954174 |
| NCT00948701 |
| NCT00946712 |
| NCT00945607 |
| NCT00942357 |
| NCT00942331 |
| NCT00929084 |
| NCT00927433 |
| NCT00925652 |
| NCT00909818 |
| NCT00904033 |
| NCT00887536 |
| NCT00877500 |
| NCT00876395 |
| NCT00869206 |
| NCT00861705 |
| NCT00856492 |
| NCT00841828 |
| NCT00828139 |
| NCT00789581 |
| NCT00785291 |
| NCT00770809 |
| NCT00769470 |
| NCT00769379 |
| NCT00721409 |
| NCT00698815 |
| NCT00684983 |
| NCT00656786 |
| NCT00644228 |
| NCT00630032 |
| NCT00613275 |
| NCT00602459 |
| NCT00601900 |
| NCT00588770 |
| NCT00581256 |
| NCT00569127 |
| NCT00567580 |
| NCT00567554 |
| NCT00556374 |
| NCT00556218 |
| NCT00553410 |
| NCT00553358 |
| NCT00532727 |
| NCT00530868 |
| NCT00524277 |
| NCT00520975 |
| NCT00513292 |
| NCT00507923 |
| NCT00499603 |
| NCT00493870 |
| NCT00486668 |
| NCT00470236 |
| NCT00463489 |
| NCT00454805 |
| NCT00453154 |
| NCT00451555 |
| NCT00433589 |
| NCT00433511 |
| NCT00433420 |
| NCT00432172 |
| NCT00424164 |
| NCT00416754 |
| NCT00410813 |
| NCT00408408 |
| NCT00390455 |
| NCT00390169 |
| NCT00382070 |
| NCT00382018 |
| NCT00378703 |
| NCT00377156 |
| NCT00376597 |
| NCT00365105 |
| NCT00355381 |
| NCT00345072 |
| NCT00337103 |
| NCT00331773 |
| NCT00326898 |
| NCT00324805 |
| NCT00321048 |
| NCT00313170 |
| NCT00310180 |
| NCT00301925 |
| NCT00301457 |
| NCT00295620 |
| NCT00294996 |
| NCT00289263 |
| NCT00287898 |
| NCT00282035 |
| NCT00274469 |
| NCT00265941 |
| NCT00265850 |
| NCT00253422 |
| NCT00248287 |
| NCT00229697 |
| NCT00217737 |
| NCT00204490 |
| NCT00204477 |
| NCT00203372 |
| NCT00186602 |
| NCT00134056 |
| NCT00130533 |
| NCT00129389 |
| NCT00126581 |
| NCT00118846 |
| NCT00103181 |
| NCT00099437 |
| NCT00096369 |
| NCT00087178 |
| NCT00086762 |
| NCT00082641 |
| NCT00079248 |
| NCT00078832 |
| NCT00077168 |
| NCT00077090 |
| NCT00075764 |
| NCT00074152 |
| NCT00072462 |
| NCT00072293 |
| NCT00072020 |
| NCT00070564 |
| NCT00070499 |
| NCT00066703 |
| NCT00066690 |
| NCT00063882 |
| NCT00053898 |
| NCT00052910 |
| NCT00052156 |
| NCT00052130 |
| NCT00045032 |
| NCT00041119 |
| NCT00039546 |
| NCT00033683 |
| NCT00030758 |
| NCT00025688 |
| NCT00005970 |
| NCT00005957 |
| NCT00005886 |
| NCT00005581 |
| NCT00004935 |
| NCT00004179 |
| NCT00004067 |
| NCT00003857 |
| NCT00003680 |
| NCT00003679 |
| NCT00003678 |
| NCT00003612 |
| NCT00003418 |
| NCT00002851 |
| NCT00001806 |
| NCT02524561 |
| NCT02450058 |
| NCT02403973 |
| NCT02382094 |
| NCT02337582 |
| NCT02270580 |
| NCT02234479 |
| NCT02223091 |
| NCT02202252 |
| NCT02142959 |
| NCT02056483 |
| NCT02056067 |
| NCT02010008 |
| NCT01985724 |
| NCT01977599 |
| NCT01945476 |
| NCT01871948 |
| NCT01830933 |
| NCT01824498 |
| NCT01815294 |
| NCT01805089 |
| NCT01752907 |
| NCT01724866 |
| NCT01723943 |
| NCT01706627 |
| NCT01703754 |
| NCT01688479 |
| NCT01674881 |
| NCT01672684 |
| NCT01669733 |
| NCT01647594 |
| NCT01642628 |
| NCT01617668 |
| NCT01613352 |
| NCT01596179 |
| NCT01577420 |
| NCT01569672 |
| NCT01569087 |
| NCT01559194 |
| NCT01558258 |
| NCT01540955 |
| NCT01535040 |
| NCT01528345 |
| NCT01519700 |
| NCT01468766 |
| NCT01468675 |
| NCT01466270 |
| NCT01439945 |
| NCT01439191 |
| NCT01434264 |
| NCT01421004 |
| NCT01419613 |
| NCT01396174 |
| NCT01387841 |
| NCT01385137 |
| NCT01384838 |
| NCT01383174 |
| NCT01373320 |
| NCT01372527 |
| NCT01369173 |
| NCT01361035 |
| NCT01339351 |
| NCT01300351 |
| NCT01282580 |
| NCT01267877 |
| NCT01256008 |
| NCT01240213 |
| NCT01234506 |
| NCT01225172 |
| NCT01217333 |
| NCT01217216 |
| NCT01202591 |
| NCT01186796 |
| NCT01177124 |
| NCT01171924 |
| NCT01156753 |
| NCT01129336 |
| NCT01116713 |
| NCT01108016 |
| NCT01106820 |
| NCT01089933 |
| NCT01086683 |
| NCT01079923 |
| NCT01073774 |
| NCT01048606 |
| NCT01048528 |
| NCT01043003 |
| NCT01038804 |
| NCT01004081 |
| NCT01003158 |
| NCT00994279 |
| NCT00992706 |
| NCT00990977 |
| NCT00983684 |
| NCT00982319 |
| NCT00976365 |
| NCT00966719 |
| NCT00963729 |
| NCT00962494 |
| NCT00956813 |
| NCT00951054 |
| NCT00944918 |
| NCT00940225 |
| NCT00938652 |
| NCT00933309 |
| NCT00932997 |
| NCT00931008 |
| NCT00930930 |
| NCT00929240 |
| NCT00915603 |
| NCT00903474 |
| NCT00900627 |
| NCT00864253 |
| NCT00863655 |
| NCT00827580 |
| NCT00802945 |
| NCT00775645 |
| NCT00754325 |
| NCT00742755 |
| NCT00712647 |
| NCT00710658 |
| NCT00702949 |
| NCT00698035 |
| NCT00696072 |
| NCT00693992 |
| NCT00690196 |
| NCT00688740 |
| NCT00687102 |
| NCT00676663 |
| NCT00674830 |
| NCT00672217 |
| NCT00670878 |
| NCT00668174 |
| NCT00662103 |
| NCT00656305 |
| NCT00639366 |
| NCT00626106 |
| NCT00617708 |
| NCT00615940 |
| NCT00615602 |
| NCT00612443 |
| NCT00609505 |
| NCT00605267 |
| NCT00593827 |
| NCT00590213 |
| NCT00574873 |
| NCT00572416 |
| NCT00565305 |
| NCT00558272 |
| NCT00555386 |
| NCT00545077 |
| NCT00540358 |
| NCT00537771 |
| NCT00530764 |
| NCT00525980 |
| NCT00522262 |
| NCT00519545 |
| NCT00513916 |
| NCT00513136 |
| NCT00511459 |
| NCT00497809 |
| NCT00496678 |
| NCT00494481 |
| NCT00493636 |
| NCT00489411 |
| NCT00486525 |
| NCT00478257 |
| NCT00477646 |
| NCT00476203 |
| NCT00475085 |
| NCT00473096 |
| NCT00470561 |
| NCT00470119 |
| NCT00469339 |
| NCT00467844 |
| NCT00467493 |
| NCT00463788 |
| NCT00462891 |
| NCT00461526 |
| NCT00455533 |
| NCT00450892 |
| NCT00440089 |
| NCT00438659 |
| NCT00438100 |
| NCT00436566 |
| NCT00435409 |
| NCT00431106 |
| NCT00431080 |
| NCT00429871 |
| NCT00427245 |
| NCT00424606 |
| NCT00416403 |
| NCT00405938 |
| NCT00393939 |
| NCT00393172 |
| NCT00388726 |
| NCT00385515 |
| NCT00382083 |
| NCT00379782 |
| NCT00375024 |
| NCT00373256 |
| NCT00370552 |
| NCT00363909 |
| NCT00357110 |
| NCT00343382 |
| NCT00336791 |
| NCT00327769 |
| NCT00325234 |
| NCT00322348 |
| NCT00320710 |
| NCT00319618 |
| NCT00312208 |
| NCT00311636 |
| NCT00305448 |
| NCT00304850 |
| NCT00303524 |
| NCT00300508 |
| NCT00299286 |
| NCT00296036 |
| NCT00295529 |
| NCT00293540 |
| NCT00291759 |
| NCT00288795 |
| NCT00287534 |
| NCT00287040 |
| NCT00286117 |
| NCT00281957 |
| NCT00270166 |
| NCT00266799 |
| NCT00262899 |
| NCT00262847 |
| NCT00259090 |
| NCT00256698 |
| NCT00255463 |
| NCT00252421 |
| NCT00248170 |
| NCT00247975 |
| NCT00247481 |
| NCT00246571 |
| NCT00241449 |
| NCT00239343 |
| NCT00233610 |
| NCT00232661 |
| NCT00230711 |
| NCT00225927 |
| NCT00221221 |
| NCT00220792 |
| NCT00214162 |
| NCT00211133 |
| NCT00209092 |
| NCT00201929 |
| NCT00201890 |
| NCT00201851 |
| NCT00200174 |
| NCT00188305 |
| NCT00186524 |
| NCT00182234 |
| NCT00175344 |
| NCT00170235 |
| NCT00167674 |
| NCT00164658 |
| NCT00150917 |
| NCT00148057 |
| NCT00144898 |
| NCT00129935 |
| NCT00129922 |
| NCT00128843 |
| NCT00128778 |
| NCT00128310 |
| NCT00128297 |
| NCT00122369 |
| NCT00121992 |
| NCT00121160 |
| NCT00115713 |
| NCT00107497 |
| NCT00107016 |
| NCT00096356 |
| NCT00093002 |
| NCT00091442 |
| NCT00087399 |
| NCT00083304 |
| NCT00082433 |
| NCT00082277 |
| NCT00080301 |
| NCT00079274 |
| NCT00077025 |
| NCT00068458 |
| NCT00068341 |
| NCT00067691 |
| NCT00066586 |
| NCT00066378 |
| NCT00065325 |
| NCT00062751 |
| NCT00060320 |
| NCT00055991 |
| NCT00054561 |
| NCT00054418 |
| NCT00052351 |
| NCT00050427 |
| NCT00050141 |
| NCT00050011 |
| NCT00049465 |
| NCT00046891 |
| NCT00046865 |
| NCT00045292 |
| NCT00044291 |
| NCT00037999 |
| NCT00032136 |
| NCT00031772 |
| NCT00028990 |
| NCT00026117 |
| NCT00024102 |
| NCT00022087 |
| NCT00022074 |
| NCT00021255 |
| NCT00019916 |
| NCT00017095 |
| NCT00016432 |
| NCT00016406 |
| NCT00014612 |
| NCT00014222 |
| NCT00008411 |
| NCT00008385 |
| NCT00006031 |
| NCT00005980 |
| NCT00005975 |
| NCT00005879 |
| NCT00005865 |
| NCT00005590 |
| NCT00005588 |
| NCT00005587 |
| NCT00004125 |
| NCT00003992 |
| NCT00003991 |
| NCT00003972 |
| NCT00003938 |
| NCT00003906 |
| NCT00003884 |
| NCT00003883 |
| NCT00003855 |
| NCT00003830 |
| NCT00003782 |
| NCT00003771 |
| NCT00003752 |
| NCT00003730 |
| NCT00003674 |
| NCT00003600 |
| NCT00003519 |
| NCT00003351 |
| NCT00003279 |
| NCT00003162 |
| NCT00003140 |
| NCT00003125 |
| NCT00003088 |
| NCT00003032 |
| NCT00003013 |
| NCT00003010 |
| NCT00002937 |
| NCT00002920 |
| NCT00002784 |
| NCT00002777 |
| NCT00002707 |
| NCT00002646 |
| NCT00002644 |
| NCT00002582 |
| NCT00002564 |
| NCT00002542 |
| NCT02473003 |
| NCT02076958 |
| NCT01726127 |
| NCT01464970 |
| NCT01315015 |
| NCT02538484 |
| NCT02526719 |
| NCT02521961 |
| NCT02521623 |
| NCT02514681 |
| NCT02501031 |
| NCT02492607 |
| NCT02489409 |
| NCT02473042 |
| NCT02429544 |
| NCT02404051 |
| NCT02360059 |
| NCT02339779 |
| NCT02333890 |
| NCT02286778 |
| NCT02276443 |
| NCT02165696 |
| NCT02133703 |
| NCT01803139 |
| NCT01795404 |
| NCT01071109 |
| NCT00910884 |
| NCT02539355 |
| NCT02531997 |
| NCT02530411 |
| NCT02529280 |
| NCT02524548 |
| NCT02518477 |
| NCT02516540 |
| NCT02513407 |
| NCT02510781 |
| NCT02506790 |
| NCT02506777 |
| NCT02498600 |
| NCT02491983 |
| NCT02488967 |
| NCT02483923 |
| NCT02482753 |
| NCT02479607 |
| NCT02462226 |
| NCT02457910 |
| NCT02451735 |
| NCT02448420 |
| NCT02447211 |
| NCT02445391 |
| NCT02443493 |
| NCT02441946 |
| NCT02438722 |
| NCT02437318 |
| NCT02430948 |
| NCT02423603 |
| NCT02422615 |
| NCT02415387 |
| NCT02413008 |
| NCT02401685 |
| NCT02394496 |
| NCT02387320 |
| NCT02376985 |
| NCT02374099 |
| NCT02340845 |
| NCT02332876 |
| NCT02311933 |
| NCT02311543 |
| NCT02309177 |
| NCT02306161 |
| NCT02305498 |
| NCT02305173 |
| NCT02301988 |
| NCT02297698 |
| NCT02296801 |
| NCT02289898 |
| NCT02288169 |
| NCT02282020 |
| NCT02278120 |
| NCT02275754 |
| NCT02273973 |
| NCT02273206 |
| NCT02272790 |
| NCT02266017 |
| NCT02249208 |
| NCT02246621 |
| NCT02244593 |
| NCT02243007 |
| NCT02240836 |
| NCT02240199 |
| NCT02238509 |
| NCT02229539 |
| NCT02229149 |
| NCT02229136 |
| NCT02226107 |
| NCT02225015 |
| NCT02223052 |
| NCT02215668 |
| NCT02204098 |
| NCT02203552 |
| NCT02198690 |
| NCT02196181 |
| NCT02194387 |
| NCT02193282 |
| NCT02192333 |
| NCT02191982 |
| NCT02187991 |
| NCT02187744 |
| NCT02185859 |
| NCT02185352 |
| NCT02177695 |
| NCT02173262 |
| NCT02169011 |
| NCT02167490 |
| NCT02164916 |
| NCT02162329 |
| NCT02161900 |
| NCT02159157 |
| NCT02158962 |
| NCT02158832 |
| NCT02154490 |
| NCT02152137 |
| NCT02148575 |
| NCT02144194 |
| NCT02140437 |
| NCT02137252 |
| NCT02136927 |
| NCT02134912 |
| NCT02125006 |
| NCT02115282 |
| NCT02115048 |
| NCT02107703 |
| NCT02101788 |
| NCT02101385 |
| NCT02096588 |
| NCT02085408 |
| NCT02079662 |
| NCT02077569 |
| NCT02066181 |
| NCT02065687 |
| NCT02062255 |
| NCT02051218 |
| NCT02048813 |
| NCT02037529 |
| NCT02032823 |
| NCT02028507 |
| NCT02028494 |
| NCT02015559 |
| NCT02006979 |
| NCT02004496 |
| NCT02003222 |
| NCT02003209 |
| NCT02002533 |
| NCT02000622 |
| NCT01997333 |
| NCT01989780 |
| NCT01989676 |
| NCT01988571 |
| NCT01982448 |
| NCT01975363 |
| NCT01975064 |
| NCT01963572 |
| NCT01958021 |
| NCT01954641 |
| NCT01953588 |
| NCT01950390 |
| NCT01948128 |
| NCT01945775 |
| NCT01934335 |
| NCT01929395 |
| NCT01928394 |
| NCT01927081 |
| NCT01926678 |
| NCT01923168 |
| NCT01921335 |
| NCT01905592 |
| NCT01904266 |
| NCT01903811 |
| NCT01902745 |
| NCT01901146 |
| NCT01901094 |
| NCT01900418 |
| NCT01893112 |
| NCT01886872 |
| NCT01879878 |
| NCT01875367 |
| NCT01872975 |
| NCT01872260 |
| NCT01871116 |
| NCT01866670 |
| NCT01864746 |
| NCT01857193 |
| NCT01853748 |
| NCT01841736 |
| NCT01840163 |
| NCT01835236 |
| NCT01835145 |
| NCT01824875 |
| NCT01823991 |
| NCT01816594 |
| NCT01815346 |
| NCT01814813 |
| NCT01811264 |
| NCT01809691 |
| NCT01808573 |
| NCT01805076 |
| NCT01803958 |
| NCT01802749 |
| NCT01802333 |
| NCT01792726 |
| NCT01792050 |
| NCT01789684 |
| NCT01786122 |
| NCT01772472 |
| NCT01764802 |
| NCT01758146 |
| NCT01725633 |
| NCT01708798 |
| NCT01706081 |
| NCT01704586 |
| NCT01674140 |
| NCT01670500 |
| NCT01668719 |
| NCT01656538 |
| NCT01635413 |
| NCT01633060 |
| NCT01625286 |
| NCT01622868 |
| NCT01598298 |
| NCT01597414 |
| NCT01575548 |
| NCT01573442 |
| NCT01570036 |
| NCT01560416 |
| NCT01548677 |
| NCT01544374 |
| NCT01535066 |
| NCT01535053 |
| NCT01533207 |
| NCT01527409 |
| NCT01526135 |
| NCT01521000 |
| NCT01515787 |
| NCT01509781 |
| NCT01471106 |
| NCT01452672 |
| NCT01419730 |
| NCT01415752 |
| NCT01349881 |
| NCT01339000 |
| NCT01329068 |
| NCT01318005 |
| NCT01310231 |
| NCT01310075 |
| NCT01299623 |
| NCT01276704 |
| NCT01274338 |
| NCT01272037 |
| NCT01242800 |
| NCT01231802 |
| NCT01226316 |
| NCT01202851 |
| NCT01196936 |
| NCT01196390 |
| NCT01186367 |
| NCT01169337 |
| NCT01160211 |
| NCT01150045 |
| NCT01120249 |
| NCT01104571 |
| NCT01101451 |
| NCT01100463 |
| NCT01089764 |
| NCT01088893 |
| NCT01034215 |
| NCT01016886 |
| NCT01013649 |
| NCT00984321 |
| NCT00966888 |
| NCT00944424 |
| NCT00914017 |
| NCT00896155 |
| NCT00892814 |
| NCT00892515 |
| NCT00885183 |
| NCT00842998 |
| NCT00818051 |
| NCT00814567 |
| NCT00685256 |
| NCT00671476 |
| NCT00651417 |
| NCT00637325 |
| NCT00565851 |
| NCT00515411 |
| NCT00502684 |
| NCT00458796 |
| NCT00415285 |
| NCT00338286 |
| NCT00268476 |
| NCT00262184 |
| NCT00117663 |
| NCT02322853 |
| NCT02194829 |
| NCT01922921 |
| NCT01856192 |
| NCT01641068 |
| NCT01824823 |
| NCT01816555 |
| NCT01744821 |
| NCT01596530 |
| NCT01563588 |
| NCT01526369 |
| NCT01472445 |
| NCT01351844 |
| NCT01345630 |
| NCT01262274 |
| NCT01259284 |
| NCT01151215 |
| NCT01050075 |
| NCT01011478 |
| NCT00959985 |
| NCT00925548 |
| NCT00908531 |
| NCT00903006 |
| NCT00802711 |
| NCT00777335 |
| NCT00770354 |
| NCT00752986 |
| NCT00691119 |
| NCT00674557 |
| NCT00662597 |
| NCT00657137 |
| NCT00625898 |
| NCT00555841 |
| NCT00543127 |
| NCT00536081 |
| NCT00522392 |
| NCT00516269 |
| NCT00503581 |
| NCT00499369 |
| NCT00453635 |
| NCT00440622 |
| NCT00437359 |
| NCT00431795 |
| NCT00373113 |
| NCT00372996 |
| NCT00337272 |
| NCT00305643 |
| NCT00303628 |
| NCT00282529 |
| NCT00281528 |
| NCT00251095 |
| NCT00183963 |
| NCT00165880 |
| NCT00132574 |
| NCT00130507 |
| NCT00130494 |
| NCT00097344 |
| NCT00090844 |
| NCT00089141 |
| NCT00083993 |
| NCT00082095 |
| NCT00049660 |
| NCT00045591 |
| NCT00036686 |
| NCT00028704 |
| NCT00009906 |
| NCT00004893 |
| NCT00002668 |
